# The Genetic Diversity and Population Structure of the Endemic *Alyssum bosniacum* (Brassicaceae) from the Central Dinaric Alps, Balkan Peninsula

**DOI:** 10.3390/plants15020224

**Published:** 2026-01-11

**Authors:** Jasna Hanjalić Kurtović, Belma Kalamujić Stroil, Naris Pojskić, Sonja Siljak-Yakovlev, Alma Hajrudinović-Bogunić, Faruk Bogunić

**Affiliations:** 1 Institute for Genetic Engineering and Biotechnology, University of Sarajevo, Zmaja od Bosne 8, 71 000 Sarajevo, Bosnia and Herzegovina; jasna.hanjalic@ingeb.unsa.ba (J.H.K.); naris.pojskic@ingeb.unsa.ba (N.P.); 2 Ecologie Société Evolution, CNRS, AgroParisTech, Université Paris-Saclay, 91190 Gif-sur-Yvette, France; 3 Faculty of Forestry, University of Sarajevo, Zagrebačka 20, 71 000 Sarajevo, Bosnia and Herzegovina; a.hajrudinovic@sfsa.unsa.ba (A.H.-B.); f.bogunic@sfsa.unsa.ba (F.B.)

**Keywords:** *Alyssum*, Balkans, high mountains, endemic, diversity, structure

## Abstract

The Balkan mountain ranges are major hotspots of genetic diversity and endemism, yet many species remain poorly studied. One such species is *Alyssum bosniacum*, a narrow endemic of the Central Dinaric Alps. To fill this gap, we examined 143 individuals from 15 populations across the species’ range using flow-cytometric ploidy determination, amplified fragment length polymorphisms (AFLPs), nuclear microsatellites, and chloroplast DNA sequences. Microsatellite data revealed two genetic clusters, showing moderate differentiation and relatively high diversity. AFLP profiles indicated shallow but geographically structured variation, while chloroplast haplotypes showed limited divergence and regional clustering. Our data suggest possible persistence in multiple microrefugia within the Central Dinaric Alps, although further evidence is needed to confirm this scenario. Despite range fragmentation, genetic variation within the population remains high, indicating evolutionary resilience and supporting the species’ long-term future population stability under current conditions.

## 1. Introduction

The Balkan peninsula is one of the major European biodiversity hotspots, characterized by exceptional plant diversity driven by complex topography, climatic history, substrate heterogeneity, and long-term anthropogenic influence [1,2,3,4]. It represents a unique reservoir of floristic and genetic diversity, harbouring numerous endemic and relic species, and diverse infraspecific lineages [5,6,7]. During the Pleistocene, the Balkans served as an important refugium [8,9] and a source of postglacial recolonisation of Central Europe [10,11,12,13], the Apennine Peninsula [14,15], and the Carpathians [14,16]. The repeated glacial–interglacial cycles shaped latitudinal and altitudinal range dynamics of many species [8,17,18].

Conversely, many plant species did not expand their distribution ranges beyond the Balkans, resulting in regionally intraspecific diversification [19,20]. This pattern is particularly pronounced in high-alpine endemics, where the complex mountain topography promotes spatial isolation and intraspecific genetic differentiation [5,21]. Although the extent of glaciation during the Last Glacial Maximum was significantly lower in the Balkans [22,23] compared to the Alps [24], diversification of many endemic species remains confined to Balkan mountain systems [25,26,27,28,29,30].

*Alyssum* L. is among the genera characterized by high taxonomic complexity in the Balkan Peninsula [6]. Comprehensive studies of the polyploid *Alyssum montanum*-*A. repens* complex have revealed exceptional genetic and morphological variation within the genus, often geographically structured and shaped by frequent auto- and allopolyploidization events, indicating a highly complex evolutionary history [6,31]. While most species are found at lower elevations, only a few occur in strictly high-mountain habitats (*A. bosniacum* Beck, *A. handelii* Hayek, *A. pirinicum* (Stoj. & Acht.) Ančev. [6,31].

*Alyssum bosniacum* is an endemic species of the mountainous systems of the Central Dinaric Alps, with its entire distribution confined to Bosnia and Herzegovina [31]. It occurs in small, isolated populations across a fragmented high-mountain range [32], typically inhabiting carbonate rocky terrains, alpine meadows, and rock crevices [31,32,33]. Four populations of the species have been previously investigated using molecular markers, flow cytometry, and morphometrics to clarify its phylogenetic position with other Balkan taxa [6,31]. These studies revealed heteroploid populations and close relationships with the steno-endemic *A. moellendorfianum* Asch. ex Beck, which is found at lower altitudes [6]. Considering the relatively limited distribution across the Central Dinaric mountain massifs, there is a notable lack of data not only on the spatial distribution of ploidy, genetic diversity, and structure, but also on the species’ habitats and its precise overall distribution.

Thus, as a first step in studying *A. bosniacum*, we aimed to assess genetic diversity, population structure, and relationships among its populations using a multi-marker approach. We sampled 15 populations covering its whole range in the Central Dinaric Alps and performed analyses using nuclear microsatellites, amplified fragment length polymorphisms (AFLPs), chloroplast DNA sequences, and flow cytometric ploidy determination.

All sampling sites of the studied populations were located in mountain areas that were glaciated during the Last Glacial Maximum [34,35]. Based on previous studies on Balkan *Alyssum* [6,30,31], we hypothesize that spatial isolations due to mountain topography, accompanied by polyploidy, primarily shapes the genetic diversity and population structure of the species within the Central Dinaric Alps. Accordingly, we expect nuclear markers to reveal pronounced population structuring among isolated populations, whereas plastid markers will exhibit lower diversity and weaker spatial structure.

## 2. Results

### 2.1. Genome Size and Ploidy Variation

**Figure 1:**
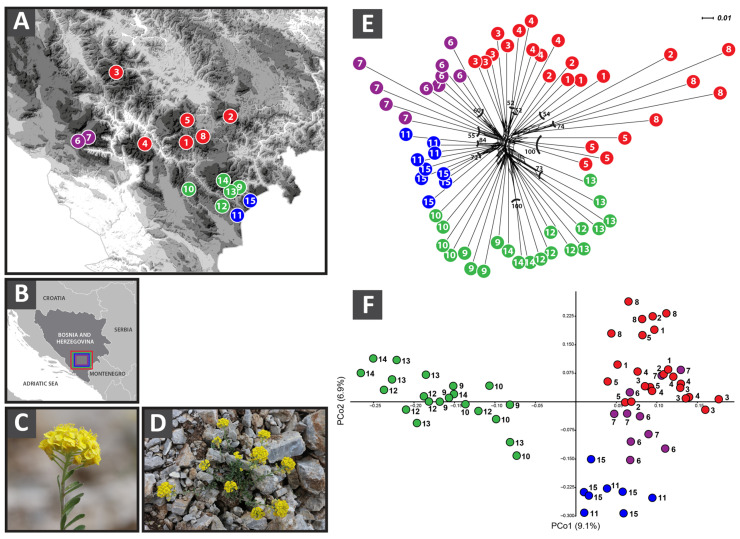
(**A**) Sampling sites of *Alyssum bosniacum* populations analyzed in the present study. Population numbers correspond to those given in the subsequent tables. Colors of the sampled locations correspond to clusters in the NeighborNet diagram, (**B**) Species distribution range in Bosnia and Herzegovina (Western Balkans), (**C**,**D**) Habitus of *A. bosniacum*; photo: F. Bogunić. (**E**) NeighborNet based on AFLP data within *A. bosniacum* accessions supplemented with bootstrap values ≥50% derived from a Neighbor-joining analysis. (**F**) Principal coordinate analysis (PCoA) of Dice distances among *Alyssum bosniacum* accessions based on AFLP data.

The flow cytometric measurements of *A. bosniacum* (143 plants/15 populations) revealed the two cytotypes, diploid and tetraploid, with the tetraploid detected only in Population 2 (Figure 1A, Table 1). No intraploid variation was detected within the studied populations. Diploid cytotypes exhibited mean absolute (2C) genome sizes ranging from 1.30 pg to 1.50 pg, while the tetraploid cytotype measured 2.74 pg (Table 1). Monoploid (1Cx) genome size values ranged from 0.64 pg to 0.75 pg. The coefficient of variation per population ranged from 1.97% to 4.6%, except for populations 3 (5.08%) and 7 (6.74%) (Table 1).

Mean 1Cx values statistically differed among *A. bosniacum* populations (F_14, 140_ = 12.01; *p* ≤ 0.001). Tukey’s test identified significant differences between populations (*p* ≤ 0.05, Table 2), with Populations 3, 4 and 7 differing from most others (P3 vs. P4–6, 8–15; P4 vs. P9, 11–13, 15; P7 vs. P9, 11–13, 15).

**Table 1:** Absolute (2C) and monoploid (1Cx) genome size of *Alyssum bosniacum*.

			2C (pg)			1Cx (pg)			1Cx (Mbp)
Population Number	*N_FCM_*	Ploidy	Mean (pg) ± SD	Min–Max	CV (%)	Mean (pg) ± SD	Min–Max	CV (%)	Mean (pg) ± SD
1	10	2×	1.39 ± 0.02	1.36–1.43	1.97	0.69 ± 0.01	0.68–0.71	1.79	681.86 ± 12.54
2	10	4×	2.74 ± 0.11	2.56–2.86	4.06	0.68 ± 0.02	0.64–0.71	4.13	670.46 ± 27.25
3	10	2×	1.50 ± 0.07	1.40–1.63	5.19	0.75 ± 0.03	0.70–0.81	5.08	735.25 ± 38.03
4	9	2×	1.40 ± 0.05	1.33–1.49	3.69	0.70 ± 0.02	0.67–0.75	3.75	688.18 ± 25.81
5	10	2×	1.37 ± 0.04	1.32–1.48	3.02	0.68 ± 0.02	0.66–0.74	3.09	669.42 ± 19.92
6	9	2×	1.38 ± 0.04	1.32–1.44	3.37	0.69 ± 0.02	0.66–0.72	3.06	678.27 ± 22.75
7	8	2×	1.45 ± 0.10	1.34–1.62	6.88	0.72 ± 0.04	0.67–0.81	6.74	711.20 ± 49.24
8	8	2×	1.34 ± 0.06	1.27–1.44	4.61	0.67 ± 0.03	0.63–0.72	4.75	657.78 ± 30.72
9	10	2×	1.30 ± 0.04	1.21–1.38	3.60	0.64 ± 0.02	0.60–0.69	3.73	636.83 ± 23.50
10	10	2×	1.37 ± 0.04	1.30–1.43	3.02	0.68 ± 0.02	0.65–0.71	3.09	670.64 ± 20.44
11	19	2×	1.32 ± 0.05	1.23–1.45	3.92	0.66 ± 0.02	0.61–0.73	4.07	646.84 ± 25.61
12	10	2×	1.30 ± 0.03	1.23–1.36	2.70	0.65 ± 0.01	0.62–0.68	2.48	637.52 ± 17.12
13	10	2×	1.31 ± 0.04	1.28–1.44	3.50	0.65 ± 0.02	0.64–0.72	3.46	644.88 ± 21.71
14	10	2×	1.35 ± 0.03	1.30–1.40	2.53	0.67 ± 0.01	0.65–0.70	2.61	661.64 ± 15.95
15	10	2×	1.37 ± 0.04	1.32–1.45	3.49	0.68 ± 0.02	0.66–0.72	3.49	673.35 ± 23.00

2×—diploid; 4×—tetraploid; SD—standard deviation; CV—coefficient of variation; Min–Max—minimal and maximal values for 2C and 1Cx genome size. Population codes are consistently used in all tables and figures.

**Table 2:** Average values of genetic diversity parameters for four analyzed loci per population. * tetraploid population.

Population Number	A_N_	A_E_	AR	R	*p*(R)	H_E_	H_O_	F
1	4.50	2.69	4.50	0.597	0.290	0.542	0.487	0.209
2 *	4.75	3.34	4.75	0.698	0.415	0.617	0.637	0.192
3	3.75	3.01	3.75	0.793	0.756	0.604	0.587	0.028
4	4.25	2.45	4.25	0.574	0.268	0.542	0.363	0.261
5	3.75	2.72	3.75	0.678	0.559	0.539	0.250	0.491
6	4.00	2.19	4.00	0.627	0.339	0.539	0.550	−0.053
7	4.00	2.47	4.00	0.711	0.526	0.588	0.563	0.012
8	4.25	2.90	4.25	0.660	0.484	0.582	0.463	0.137
9	4.75	2.89	4.75	0.635	0.399	0.591	0.337	0.343
10	4.25	2.80	4.25	0.627	0.458	0.572	0.475	0.226
11	4.25	2.96	4.25	0.752	0.543	0.587	0.475	0.131
12	3.75	2.75	3.75	0.734	0.702	0.548	0.463	0.160
13	3.75	2.38	3.75	0.645	0.432	0.572	0.475	0.156
14	5.25	3.50	5.25	0.671	0.400	0.689	0.525	0.188
15	5.25	3.45	5.25	0.656	0.539	0.637	0.588	0.061

A_N_—number of detected alleles; A_E_—number of effective alleles; AR—allelic richness; R—the effective and detected number of alleles ratio; *p*(R)—statistical significance at *p* < 0.05; H_E_—expected heterozygosity; H_O_—observed heterozygosity; F—inbreeding coefficient.

### 2.2. Microsatellite Analyses

Average values of genetic diversity measures across four microsatellite loci per population are presented in Table 2, with locus-specific data available in Supplementary Table S1. Populations 14 and 15 exhibited the highest allelic richness (AR = 5.25) and the greatest average number of alleles (A_N_), as well as the highest effective number of alleles (A_E_ = 3.50 and 3.45, respectively). In contrast, populations 3, 5, 12, and 13 showed the lowest A_N_ and AR (AR = 3.75), while Population 6 had the lowest A_E_ (2.19).

Statistically significant (*p* < 0.05) differences in the ratio of effective to detected alleles (R) were observed at locus AP31679 in Population 14 (*p* = 0.023) and at locus AP10368 in five populations: 6, 7, 9, 11, and 15 (Supplementary Table S1). The ratio (R) between the number of effective alleles and detected alleles (A_E_/A_N_) reflects the potential statistical significance of differences between these two indicators of genetic diversity. This metric shows whether the effective allele number—representing alleles that actually contribute to genetic diversity—differs significantly from the total number of detected alleles. Identifying such differences can help reveal trends of allele loss at specific loci. The highest average observed heterozygosity (H_O_) was recorded in tetraploid Population 2 (0.637), while the lowest value (0.250) was observed in Population 5. Notably, some populations exhibited a decoupling between heterozygosity and allelic richness. In particular, Population 3 showed relatively high observed and expected heterozygosity (H_O_ = 0.587; H_E_ = 0.604) despite low allelic richness (AR = 3.75). This pattern contrasts with populations such as Population 5, which displayed both low heterozygosity and low allelic richness, and highlights heterogeneity in the underlying genetic processes shaping diversity among populations.

Basic heterogeneity measures per locus for all populations of *A. bosniacum* are summarized in Table 3. The R values ranged from 0.306 (AP31733) to 0.458 (AP10368), with statistically significant variation (*p* < 0.05) across all loci. High frequencies of one to two dominant alleles were consistently observed across loci (Supplementary Table S1).

The major allele frequency index (iMAF) is defined as the ratio between the expected allele frequency—assuming equal frequency of all alleles—and the highest allele frequency at a given locus. This metric illustrates the relationship between the most frequent allele and the total number of detected alleles. As one of the indicators used in genetic diversity assessment, iMAF reflects the tendency of alleles toward fixation, which can ultimately lead to the loss of less frequent alleles. iMAF showed statistical significance (*p* < 0.01) across all loci, while it was at the threshold of statistical significance (*p* = 0.018) at locus AP31679 (Table 3).

**Table 3:** Genetic diversity parameters for total sample of *Alyssum bosniacum* per loci.

Locus	A_N_	A_E_	AR	R	*p*(R)	H_E_	H_O_	F	iMAF	*p*(iMAF)	F_IT_	F_IS_	F_ST_
AP31679	8	3.36	5.85	0.420	0.022	0.702	0.583	0.166	0.315	0.018	0.172	0.186	0.123
AP31733	8	2.45	5.07	0.306	0.016	0.591	0.293	0.542	0.219	0.003	0.545	0.071	0.108
AP31640	6	2.08	4.63	0.347	0.047	0.520	0.647	−0.239	0.251	0.007	−0.230	0.471	0.141
AP10368	13	5.96	8.93	0.458	0.007	0.832	0.407	0.524	0.251	0.007	0.528	−0.392	0.116
Mean	9.00	3.46	6.12	0.382	0.023	0.661	0.482	0.282	0.259	0.008	0.287	0.186	0.123

A_N_—number of detected alleles; A_E_—number of effective alleles; AR—allelic richness; R—the effective and detected number of alleles ratio; *p*(R)—statistical significance at *p* < 0.05; H_E_—expected heterozygosity; H_O_—observed heterozygosity; F—inbreeding coefficient; iMAF—major allele frequency index; *p*(iMAF)—statistical significance at *p* < 0.01; F_IT_—the inbreeding coefficient of an individual relative to the total population. F_IS_—the inbreeding coefficient of an individual relative to its subpopulation. F_ST_—the level of genetic differentiation among subpopulations.

The average values of the fixation index (F_ST_) suggested that 12.36% of the total genetic variation corresponded to differences among 15 investigated populations, which was concordant with the analysis of molecular variance (AMOVA) that showed the total variation was due to differences within populations (98.5%).

**Figure 2:**
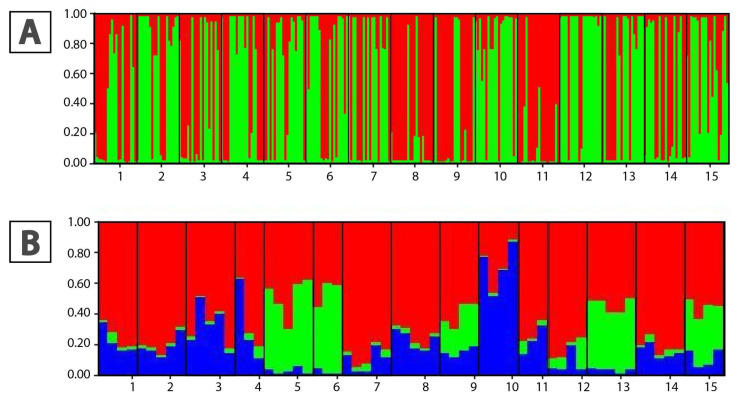
Results of STRUCTURE analysis for 15 analyzed populations of *Alyssum bosniacum*. (**A**) Proportion (%) to genetic clusters according to STRUCTURE analysis of microsatellite loci; (**B**) STRUCTURE analysis according to the AFLP matrix. Red—cluster I, green—cluster II, blue—cluster III. Population numbers correspond to those in Table 4.

Pairwise F_ST_ indicated low to moderate differentiation (Supplementary Table S2), with the highest differentiation observed between the diploid populations 6 and 7 (pF_ST_ = 0.3221). The Bayesian clustering model and STRUCTURE analysis of microsatellite loci revealed two clusters (K = 2), with nine populations showing greater genetical alignment with the second cluster based on the *qI* probabilities (probability of membership), including the tetraploid Population 2 (*qI* > 67%) (Figure 2A). The result of Principal Coordinates Analysis (PCoA) based on microsatellite genotype variants indicates a joint cluster of samples from all localities except Population 2 where a clear dispersion can be observed (Supplementary Figure S1). The percentage in the first half of Axis was 77.5%, since in the second 12.15%.

**Table 4:** List of *Alyssum bosniacum* sampled populations and number of analysed individuals.

Population Number	Locality	Latitude	Longitude	Altitude (m)	N_FCM_	N_STR_	N_AFLP_	N_cp_	Voucher Number
1	Crveni kuk, Mt. Visočica	43.582778	18.256389	1570	10	20	3	5	53505
2	Gola Jahorina, Mt. Jahorina	43.714536	18.579128	1809	10	20	3	5	53506
3	Above Prokoško jezero, Mt. Vranica	43.945397	17.756047	1856	10	20	5	5	53507
4	Borašnica, Mt. Prenj	43.574075	17.963742	1843	9	20	5	4	53508
5	Lukavac, Mt. Bjelašnica	43.694894	18.270106	1607	10	20	4	4	53509
6	Bare, Mt. Čvrsnica	43.576667	17.513611	1393	9	20	5	5	53510
7	Pločno, Mt. Čvrsnica	43.599444	17.544444	2087	8	20	5	3	53511
8	Ilijaš, Mt. Treskavica	43.611944	18.370000	1685	8	20	4	4	53512
9	Gornje bare, Mt. Zelengora	43.320608	18.606628	1522	10	20	4	4	53513
10	Surdup, Morine	43.331847	18.268428	1216	10	20	4	3	53514
11	Jagodino jezero, Mt. Lebršnik	43.206944	18.669167	1560	10	20	4	4	53515
12	Gornja bodežišta, Mt. Vukova planina	43.262517	18.528867	1340	10	20	5	4	53516
13	Pašina česma, Mt. Konjska glava	43.308017	18.568250	1383	10	20	5	3	53517
14	Jugovo jezero, Mt. Lelija	43.364400	18.531983	1727	10	20	3	4	53518
15	Trnovačko jezero, Mt. Maglić	43.249722	18.724169	1521	10	20	5	4	53519

Sample size for FCM measurements (N_FCM_), microsatellites (N_STR_), AFLPs (N_AFLP_), chloroplast region sequences (N_cp_).

### 2.3. AFLP Data

A total of 818 fragments were scored in 64 *A. bosniacum* individuals, of which 680 were polymorphic (83.12%), with an error rate of 5.93%. The Neighbor-Joining (NJ) tree displayed generally low bootstrap support, with significant values observed only for some terminal branches corresponding to single populations or a small number of individuals within populations. Similarly, the NeighborNet (Figure 1E) analysis revealed no clear hierarchical structure. Instead, populations clustered mainly according to geography: (I) Populations 9–10, 12–13, and 14; (II) Populations 11 and 15, both belonging to the eastern Bosnian mountain region; and (III) the largest cluster, comprising the central Bosnian populations (1–5, 8), which display close affinities with the westernmost populations, 6 and 7 (Figure 1E). The PCoA also indicated limited genetic divergence among populations and supported the observed pattern. Cluster I (Figure 1F) (Populations 9, 10, 12, 13, and 14) is separated along the first axis (PC1), while Cluster II (Populations 11 and 15) is distinguished along PC2 from the central Bosnian populations (1–5, 8). Populations 6 and 7 largely overlap with Cluster III.

STRUCTURE analysis identified K = 3 as the optimal number of genetic groups (Figure 2B). Cluster I predominated in most populations (1–7, 9, 11, 12, 14, 15; qI > 82%), while Cluster II was predominant in population 13, indicating a distinct genetic group, and Cluster III was associated with population 8.

### 2.4. Chloroplast DNA Analysis

**Figure 3:**
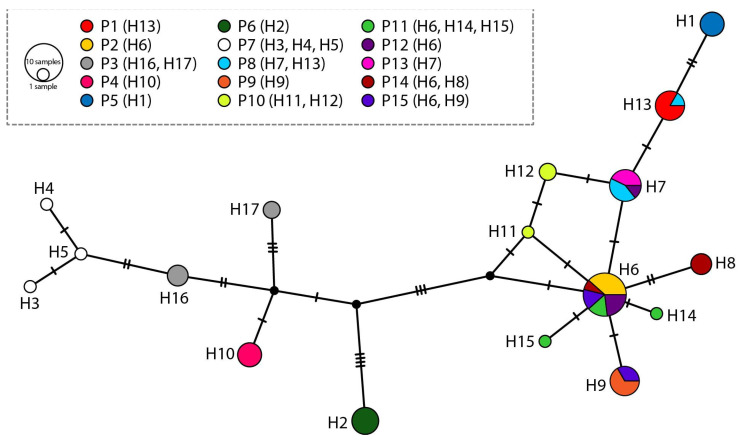
TCS haplotype network of 17 haplotypes (H1–17) found in *Alyssum bosniacum* populations. The size of each circle is proportional to the relative haplotype frequency. Black dots represent hypothetical missing haplotypes.

Amplification and sequencing of rpoB-trnC and rpl32-trnLUAG chloroplast intergenic spacers were performed for a total of 61 individuals of *A. bosniacum* (GenBank accession numbers PX564563-PX564684). A total of 17 haplotypes and 28 variable sites were detected (Supplementary Table S3). Four haplotypes (i.e., H6, H7, H9, and H13) were shared among multiple populations, while the rest were private. Except for populations 2, 4, 5, 6, 9, and 13, other populations displayed intrapopulation haplotype diversity, harbouring two or three different haplotypes. The Neighbor-Joining tree (Supplementary Figure S2) based on pairwise distances was largely congruent with the TCS haplotype network (Figure 3). Both analyses revealed low overall chloroplast divergence, reflected in short mutational distances in the parsimony network and short branch lengths in the tree. Several population-level groupings were supported by moderate to high bootstrap values in the NJ tree and corresponded to unique or shared haplotypes in the TCS network. The central haplotypes (H6 and H9) in the TCS network correspond to the internal nodes of the NJ tree. Tajima’s D was moderately negative (D = −0.915) indicating a slight excess of low-frequency mutations but not significant deviation from neutrality. In contrast, Fu’s Fs was strongly negative (Fs = −12.366), providing significant evidence for an excess of rare haplotypes.

## 3. Discussion

Our study, integrating flow cytometric ploidy estimation, microsatellite analysis, AFLP fingerprinting, and plastid DNA sequencing, revealed complex patterns of genetic variation in *A. bosniacum*. Each analysis uncovered both distinct and partially overlapping patterns of genetic variation within the species, reflecting marker-specific signatures of genetic diversity and population structure.

### 3.1. Diploid Cytotype Prevails in Populations of Alyssum bosniacum

Genome size measurements revealed an asymmetric distribution of diploid and tetraploid cytotypes in *A. bosniacum*, with the tetraploid Population 2 confined to the northeastern margin (Figure 1A). Expanding on previous studies [6,36], our sampling of additional 10 populations confirms the prevalence of the diploid cytotype within the species’ distribution range.

Some populations showed significant differences in monoploid genome size, which may reflect biological factors (the accumulation of repetitive sequences such as transposons, satellite DNA, and retroelements, absence of recombination processes, unequal homologous recombination mediated by transposons, and deletion of signals involved in double-strand DNA break repair [37,38,39,40,41]). Because silica gel-dried material was used for propidium iodide flow cytometry, an approach not generally recommended for absolute genome size determination [42], these differences should be interpreted with caution. However, our previous study using the same type of material and internal standards reported stable monoploid genome sizes among diploids and tetraploids [30]. Importantly, ploidy levels were unequivocally confirmed by our cytometric data.

Genome polyploidization in *Alyssum* comprises both allopolyploidy among shared diploid ancestors and independent autopolyploidy from a common ancestor, with Balkan taxa largely shaped by allopolyploidy [6,43,44,45]. Our results confirm the tetraploid Population 2 previously reported by Španiel et al. [6], while additional tetraploid cytotypes from Mt. Prenj (Lupoglav) and Mt. Jahorina (Pale) were documented by Magauer et al. [46] and van Loon and Kieft [47], respectively. In contrast, Population 4 (from Mt. Prenj) was identified here as diploid, originating from a different locality (Borašnica) than that examined by Magauer et al. [46]. The tetraploid cytotype from Mt. Jahorina (Pale) reported by van Loon and Kieft [47] corresponds to the area investigated for *A. bosniacum* by Španiel et al. [6]. In both earlier studies, populations were originally treated as *A. montanum* but were subsequently reassigned to *A. bosniacum* following the taxonomic revision of Balkan *Alyssum* [6]. Although diploids predominate across the species’ distribution range, previous reports [46,47] suggest that tetraploid cytotype may be underestimated and more widespread within the cytotype structure of *A. bosniacum* than currently recognized. Given the key role of polyploidy in diversification within the genus, more comprehensive and fine-scale screening of spatial ploidy distribution is needed.

The origin of tetraploid Population 2 remains unclear. Multiple analyses indicate close clustering with neighbouring populations, suggesting high genomic similarity and a possible autopolyploid origin. However, this interpretation requires further confirmation, as polyploid origins are difficult to resolve due to recurrent formation, homeologues recombination, genome restructuring, and epigenetic mechanisms across many taxa, including *Alyssum* [48].

### 3.2. Genetic Diversity and Structure of A. bosniacum

Our findings suggest that diversification and population structuring in *A. bosniacum* have primarily occurred through diploid processes, with sporadic polyploidization events. AFLP data indicate a shallow population structure largely shaped by geographic differentiation across the Central Dinaric mountain massifs, where river valleys and deep canyons likely restrict gene flow and promote population diversification [35,49]. Although diversification within Balkan *Alyssum* probably began during the Pleistocene in response to glaciation-driven range dynamics, including secondary contact of isolated populations, hybridization, and polyploidization [4,6,45], no deep genetic divergence among *A. bosniacum* populations was detected. While many studies have identified major geographic barriers shaping genetic breaks in the Dinaric Alps (e.g., the Neretva, Sutjeska, and Drina river valleys [20,50,51,52,53]), far fewer have examined genetic structure and diversity in species restricted to particular areas of this mountain range [19,51,54,55]. The same geographic barriers are present in *A. bosniacum*; however, they do not appear to have played a major role in shaping its genetic structure. This is particularly evident in Populations 6–8 from the Čvrsnica and Prenj massifs, which belong to the Mediterranean mountain system [35], and show pronounced genetic admixture with continental mountain populations (populations 1–3, 5, and 8), despite the Neretva River valley acting as a potential geographic barrier (Figure 1F). In contrast, populations 9, 10, 12, 13, and 14 from the Zelengora massif form the most divergent cluster (Figure 1E,F), even though they are geographically close to populations 11 and 15; this divergence is likely associated with isolation imposed by the Sutjeska Canyon.

During the last glacial period in the Dinaric Alps, the snowline was restricted to elevations above 1250–1500 m.a.s. [23], preventing extensive ice sheets and allowing horizontal species migrations, including *A. bosniacum*. This is consistent with STRUCTURE analysis, which identifies a single genetic cluster across ~90% of populations.

Analyses of nuclear microsatellite markers revealed a relatively uniform distribution of genetic diversity throughout the species’ range, underscoring the predominance of within-population variation. The ratio between the average number of detected alleles and effective alleles at the four loci (R) indicated that, in six populations, only a limited subset of alleles contributes significantly to overall diversity, hinting at allele fixation trends. This is supported by significant values of the index of the most abundant allele (iMAF) [56] at three loci, suggesting selective pressures acting on specific alleles.

Key parameters of genetic diversity—allelic richness and heterozygosity—provided insights into the species’ evolutionary potential. Allelic richness, reflecting long-term adaptive capacity, was relatively consistent across populations in the central part of the distribution, including the tetraploid population. Observed heterozygosity (H_O_) averaged 0.48 (range 0.25–0.63), indicating moderate to high genetic variation despite geographic isolation. An intriguing case is Population 3, which exhibits high heterozygosity but low allelic richness, suggesting strong current adaptive potential despite limited long-term variability. Conversely, low heterozygosity and allelic richness in Population 5 point to reduced adaptability. Populations 14 and 15 maintain high diversity levels, likely due to their remoteness and limited anthropogenic disturbance.

These findings align with the STRUCTURE and AMOVA analyses, which consistently show low genetic differentiation among populations, with most variation residing within rather than between them. This genetic pattern reflects ongoing gene flow and the species’ demographic history across its fragmented mountain habitats.

The haplotype network of *A. bosniacum* based on chloroplast sequences reveals a non-star-like topology, indicating a complex evolutionary history rather than a single rapid post-glacial expansion. The central position and high connectivity of H6 (Figure 3) suggest that it represents an ancestral or refugial haplotype, shared across multiple populations, while peripheral haplotypes likely evolved in isolated populations through genetic drift. The presence of intermediate haplotypes forming stepwise chains implies gradual dispersal and colonization rather than simultaneous radiation from a single source. This pattern is compatible with survival in more than one refugial area within the Central Dinaric Alps during Pleistocene climatic oscillations, followed by post-glacial recolonization of suitable habitats. Similar phylogeographic structures have been reported in other Balkan endemics, such as *Edraianthus tenuifolius* (Waldst. et Kit.) A. DC. and *Ramonda* Boiss. species, where chloroplast haplotype diversity reflects long-term persistence and isolation in microrefugia [57,58]. Molecular evidence from comparative studies supports the hypothesis of multiple refugia within the Balkans, rather than a single source of post-glacial recolonization [4,13,20,59,60].

The parsimony network also indicates that *A. bosniacum* did not experience a recent demographic expansion, which would typically produce a star-shaped topology with short branches radiating from a single ancestral haplotype [61,62]. Instead, the observed structure reflects a combination of ancient divergence and incomplete lineage sorting, possibly influenced by the species’ ecological specialization and fragmented distribution in karst habitats. This is supported by the NJ tree with short internal branches, suggesting a recent coalescence, while also revealing several geographically coherent clades that point to subsequent population structuring. The contrasting magnitude of the two neutrality statistics provides important insight into the demographic history of *A. bosniacum*. Fu’s Fs, which is highly sensitive to increases in the number of low-frequency haplotypes, yielded a strongly negative value, supporting a scenario of substantial historical population expansion. Tajima’s D, however, was only moderately negative, a pattern expected when expansion has occurred but its signal is partially obscured by subsequent geographic structuring of populations. Together, the two statistics reinforce a demographic model involving a moderate postglacial expansion from one or a few ancestral chloroplast lineages, followed by fragmentation and limited gene flow leading to the accumulation of private haplotypes in peripheral populations, consistent with the patterns observed in both the haplotype network and the NJ tree.

Comparison of genetic patterns between *A. bosniacum* and *A. moellendorfianum* [30] reveals notable differences. *Alyssum moellendorfianum*, a narrow edaphic stenoendemic at lower elevations with an estimated 250 km^2^ area of occupancy, exhibits pronounced population structure linked to ploidy, geography, and molecular markers [30]. In contrast, *A. bosniacum* shows moderate differentiation and shallow structuring, with minimal influence of polyploidization across its range. Despite their close phylogenetic relationship and overlapping distributions, the divergence of these species appears to have been driven by different mechanisms and species-specific evolutionary and historical processes. The genetic diversity and structure of *A. bosniacum* populations were likely shaped by spatial isolation due to geographic distance and barriers, combined with limited long-distance seed dispersal [45].

### 3.3. Conservation Implications

*Alyssum bosniacum* is generally not considered threatened across its overall range. However, Population 3 on Vranica mountain exhibits reduced allelic richness, while some other populations (e.g., 5 and 6) also show lower diversity or signs of allele fixation, indicating that vulnerability is not confined to a single site. Conservation priority should, therefore, be based on multiple criteria, including allelic richness, heterozygosity, population size, habitat integrity, and anthropogenic pressure. Population 3 is located near Prokoško Lake—an ecologically sensitive site subject to pronounced human-induced degradation [63]. Its habitat, located approximately 180 m above the lake, is easily accessible to hikers and visitors, increasing disturbance risk. Although the lake itself is protected under Category III and designated as a Natural Monument, enforcement of legal safeguards remains critical. Populations near areas of intense human activity and those with consistently low diversity merit heightened attention.

The urgency of natural habitats and ecosystem preservation is amplified in light of growing evidence of climate change, a major driver of distribution shifts in many high-mountain species [64,65,66,67]. The Mediterranean region, including the Central Dinaric Alps where *A. bosniacum* occurs, is particularly vulnerable to climate change according to the Regional Climate Change Index (RCCI) [68]. Given the species’ ecological specialization as a high-mountain endemic with limited dispersal capacity, climate pressures may lead to range contractions or upward elevational shifts. Such dynamics are consistent with observed genetic signatures and population fragmentation in our study and are likely to increase habitat loss risk for *A. bosniacum* and other narrowly adapted taxa [20].

Overall, moderate heterozygosity and relatively uniform diversity across most populations suggest short-term resilience. However, long-term stability may be compromised by ongoing habitat fragmentation, climate change, and localized disturbance. To safeguard evolutionary potential, we recommend: (i) genetic monitoring programs to track diversity trends and detect early signs of erosion; (ii) habitat protection and enforcement of legal safeguards, especially in tourist-accessible sites; (iii) integrated threat assessments combining genetic, ecological, and demographic data to refine conservation priorities; (iv) public awareness initiatives to mitigate anthropogenic impacts. These measures will help maintain adaptive capacity and ensure persistence of this endemic species under projected environmental changes.

## 4. Materials and Methods

### 4.1. Plant Material

During field campaigns between 2017 and 2020, a total of 15 populations of *A. bosniacum* were sampled across its entire known distribution range (Figure 1A,B). Detailed information on sampling locations and the number of analysed individuals per population is provided in Table 4. Collected fresh leaves were silica-dried and used for subsequent ploidy determination and DNA extraction. Voucher specimens were herbarized and deposited in the herbarium of the National Museum of Bosnia and Herzegovina (SARA), Sarajevo, Bosnia and Herzegovina.

### 4.2. Genome Size and Ploidy Level Determination

Absolute genome size and ploidy level were determined using flow cytometry (FCM), following the protocol of Bourge et al. [69]. Leaf tissue from *A. bosniacum* and internal standard [Lycopersicon esculentum Mill. ‘Roma’ (syn. *Solanum lycopersicum* L. ‘Montfavet 63-5’; 2C = 1.99 pg [70]) were prepared as described by Hanjalić Kurtović et al. [30]. Approximately 2000–5000 nuclei, propidium iodide-stained (final concentration 50 μg/mL, PI, Sigma Aldrich, St. Louis, MO, USA), were analysed using the CytoFLEX S flow cytometer (Beckman Coulter-Life Science, Indianapolis, IN, USA; excitation at 561 nm, 30 mW; emission detected through a 610/20 nm band-pass filter). The 2C DNA values were calculated based on the linear relationship between fluorescence intensity of the unknown samples and the internal standard. Fluorescence histograms were processed using Kaluza software ver. 2.1 (Beckman Coulter, Brea, CA, USA). Ploidy levels were inferred by comparing absolute genome size with known chromosome counts (DNA ploidy level) [71]. Standard statistical parameters, including mean value, coefficient of variation, standard deviation, and minimum and maximum values, were calculated. The normality of the genome size value distribution was assessed using the Shapiro–Wilk test. The values in Populations 5 and 13 deviated from the normality and were therefore log-transformed prior to analysis. In addition, outliers from several populations were excluded to reduce their influence on the results. Differences in the monoploid (1Cx according to [72]) values among populations were tested using one-way analysis of variance with post hoc Tukey test.

### 4.3. DNA Extraction and Molecular Analysis

Total genomic DNA was extracted from 20 mg of silica-gel dried leaf tissue using the CTAB organic extraction protocol [73,74]. Samples were homogenized using Retsch TissueLyser (Retsch GmbH, Haan, Germany). DNA quality was assessed via horizontal electrophoresis on a 1.5% agarose gel in 1X SB buffer (pH 8) [75] after staining with Midori Green Advance (Nippon Genetics Europe GmbH, Düren, Germany). Gel documentation was performed using the Vilber Fusion imaging system (Vilber Lourmat, Eberhardzell, Germany). All PCR-based procedures were conducted using GeneAmp PCR System 9700 (Applied Biosystems, Foster City, CA, USA) and Alpha Thermal Cycler (PRCmax, Cambridge, UK). Amplicons were detected using an ABI PRISM 3500 Genetic Analyser (Applied Biosystems). Capillary electrophoresis was performed under the following conditions: 50 cm capillary length, POP-7^TM^ polymer, 15.0 kV voltage, and 60 °C, with a run time of 40 min. Allele sizes were determined using the internal size standard GeneScan 500 LIZ (Applied Biosystems), and electropherograms were analysed using GeneMapper v5 software (Applied Biosystems).

### 4.4. Microsatellite Genotyping

Eight nuclear microsatellite loci developed for *Odontarrhena serpyllifolia* (Desf.) Jord. & Fourr (syn. *Alyssum serpyllifolium* Desf) from [76] were tested for cross-amplification in *A. bosniacum* (Supplementary Table S4). Amplification was performed using one singleton and two multilocus PCR reactions. The chemical and thermal conditions followed the protocol described by Hanjalić Kurtović et al. [30]. PCR products from the singleton reaction were pooled with those from Mix 2 for fragment analysis.

Complete genotypes were obtained for all samples and analysed using SPAGeDi ver. 1.5d software [77]. Expected and observed heterozygosity were calculated according to Nei [78]. Genetic diversity metrics included the effective-to-detected allele ratio (R) and the index of major allele frequency (iMAF), analysed using the R scripts ALRATIO [79] and iMAF [56]. Genetic structure and differentiation were assessed via AMOVA and F-statistics, following the method of Weir and Cockerham [80]. Population structure was inferred using a Bayesian clustering model implemented in STRUCTURE [81]. Input files were generated using the POLYSAT package [82] within R [83], treating the tetraploid population as autopolyploid and assuming polysomic inheritance. The optimal number of genetic clusters (ΔK) was estimated using Structure Harvester [84], based on the method of Evanno et al. [85]. STRUCTURE analyses were run with 100,000 burn-in generations, followed by 1,000,000 MCMC replications, across five iterations for each K value (K = 1–10). Principal Coordinates Analysis (PCoA) based on microsatellite genotypes variants was performed within the PAST v5.3 software using the Euclidean distance model as a similarity index.

### 4.5. AFLP Fingerprinting

AFLP fingerprinting was performed according to Vos et al. [86], as described in detail by Trybush et al. [87]. Three selective primer pair combinations that were used—FAM-EcoRI-A-GAG/MseI-CAC, VIC-EcoRI-A-ACG/MseI-CAC, and NED-EcoRI-A-TGC/MseI-CAC—yielded clear and polymorphic profiles. Chemical and thermal conditions followed the protocol of Hanjalić Kurtović et al. [30].

Peaks were scored using GeneMapper v5 (Applied Biosystems) to generate an automatic presence/absence genotype matrix. Amplified fragments ranging from 100 to 500 bp were scored as present (1) or absent (0) for a binary data matrix. The minimum peak detection threshold was set at 100 relative fluorescence units (rfu). All alleles were manually inspected and corrected, when necessary, based on peak width and height, with closely sized fragments assigned to the same allele category. The original dataset included 135 samples from 27 populations. Samples originating from lower-altitude populations later identified as *Alyssum austrodalmaticum* (data not published) were excluded, as they were initially collected due to the previously unknown distribution of *A. bosniacum*. Only samples from *A. bosniacum* populations, each represented by four replicates, were retained for further analyses.

Genotyping error was estimated following [88] using replicated samples, and loci with inconsistent scoring were excluded. Genetic relationships among populations were evaluated using the Nei–Li genetic distance [89]. A bootstrapped Neighbor-Joining analysis (2000 pseudoreplicates) was performed using TREECON v1.3b [90], based on uncorrected P-distances generated in SplitsTree v4.12 [91], and visualized with a neighbor-net diagram [92]. Principal Coordinates Analysis (PCoA) based on Dice distances was conducted using PAST v5.3 [93]. Population genetic structure analysis based on AFLP data was inferred using the STRUCTURE [81], applying the same parameters as used for microsatellite loci.

### 4.6. Chloroplast DNA Analyses: rpoB-trnC and rpl32-trnL^UAG^ Intergenic Spacers

Plastid haplotypes were identified using two chloroplast intergenic spacers, rpoB–trnC [94] and rpl32–trnL_UAG_ [95]. Chemical and thermal conditions followed the protocol of Hanjalić Kurtović et al. [30]. Purification and sequencing were performed by Eurofins Genomics (Ebersberg, Germany). Sequence identity was verified using BLAST 2.17.0 [96] on the NCBI GenBank platform, employing local alignment and homology search tools. Sequences were optimized in BioEdit [97], and aligned using ClustalX v2.0 [98] under default parameters (gap open penalty: 10.0; gap extension penalty: 0.2; weight matrix: Gonnet). Reference sequences of the rpl32-trnL and rpoB-trnC chloroplast regions, representing the Populations 2–5 of *A. bosniacum* from Španiel et al. [6] were used to construct the dataset for downstream analyses. Instances of heteroplasmy or ambiguous base calls remained below the limit of detection. The sequences from this study were concatenated to account for signal over noise ratio and only substitution sites were analysed. Sites with indels were treated as missing data and excluded. The concatenated matrix alignment consisted of 1650 bp positions. Plastid haplotypes were inferred using MEGA version 6.0 [99], and DNA polymorphism analysis was conducted in DNAsp v6.12.03x64 [100]. Based on concatenated sequences of rpl32–trnL and rpoB–trnC, a statistical parsimony network (TCS) was constructed using PopART v1.7 [101], following the method of Clement et al. [102], as well as a Neighbor-joining (NJ) tree based on p-distance using 1000 bootstrap replicates in MEGA software version 6.0.

## 5. Conclusions

Our study provides the first comprehensive genetic assessment of *Alyssum bosniacum* across its entire range. The most notable finding is the dominance of the diploid cytotype, with only one tetraploid population detected. Across all markers, genetic structuring is shallow, with most variation occurring within populations and only moderate differentiation among them. Chloroplast data revealed multiple haplotypes with low divergence, indicating historical connectivity rather than deep lineage splits. These results suggest that geographic isolation has shaped population structure without causing severe genetic erosion.

In summary, *A. bosniacum* maintains moderate genetic diversity and short-term resilience, but its fragmented distribution and limited dispersal capacity warrant continued monitoring. Conservation efforts should prioritize populations exposed to anthropogenic pressure and climate change to safeguard the species’ evolutionary potential.

## Data Availability

The data presented in this study are available within the article and Supplementary Materials.

## Supplementary Materials

The following supporting information can be downloaded at: https://www.mdpi.com/article/10.3390/plants15020224/s1, Table S1. Values of the genetic diversity indices for 15 studied populations of *Alyssum bosniacum* for individual microsatellite loci; Table S2. The pFST genetic differentiation matrix between 15 populations of *Alyssum bosniacum*; Table S3. Differences observed in concatenated sequences of rpl32-trnL and rpoB-trnC on the studied samples of *Alyssum bosniacum*; Table S4. Primer sequences from [76] used in this study; Figure S1. Neighbor-joining (NJ) tree based on p-distance estimates for concatenated chloroplast haplotypes of *Alyssum bosniacum*, constructed using 1000 bootstrap replicates.
