# Supplementary material for: The Genetic Diversity and Population Structure of the Endemic Alyssum bosniacum (Brassicaceae) from the Central Dinaric Alps, Balkan Peninsula
Source: Plants (Basel). 2026 Jan 11;15(2):224. doi: 10.3390/plants15020224 (PMC12845220; doi:10.3390/plants15020224)
Supplement: Supplementary file 1 [file plants-15-00224-s001.zip › plants-4043114-supplementary.pdf]

Supplementary Table S1. Values of the genetic diversity indices for 15 studied populations of *Alyssum bosniacum* for individual microsatellite loci. Population codes and numbers correspond to those in Table 4. *Tetraploid population.

Population name (code)	Locus	*N*	A_N	A_E	A_R	R	*p*(R)	H_E	H_O	F
VI (1)	AP31679	20	5	2.99	5.00	0.598	0.089	0.667	0.750	-0.129
	AP31733	20	2	1.35	2.00	0.675	0.900	0.261	0.000	1.000
	AP31640	20	4	1.84	4.00	0.460	0.090	0.458	0.550	-0.208
	AP10368	20	7	4.59	7.00	0.656	0.083	0.783	0.650	0.174
	Mean	20	4.50	2.69	4.50	0.597	0.290	0.542	0.487	0.209
JH (2)*	AP31679	20	6	4.44	6.00	0.740	0.333	0.776	0.900	-0.063
	AP31733	20	3	1.36	3.00	0.453	0.324	0.265	0.200	0.631
	AP31640	20	3	2.73	3.00	0.910	0.900	0.634	0.900	-0.299
	AP10368	20	7	4.83	7.00	0.690	0.105	0.793	0.550	0.500
	Mean	20	4.75	3.34	4.75	0.698	0.415	0.617	0.637	0.192
VRA (3)	AP31679	20	6	5.39	6.00	0.898	0.900	0.815	0.650	0.207
	AP31733	20	2	1.69	2.00	0.845	0.900	0.409	0.250	0.395
	AP31640	20	3	2.34	3.00	0.780	0.900	0.573	0.950	-0.687
	AP10368	20	4	2.61	4.00	0.652	0.325	0.618	0.500	0.195
	Mean	20	3.75	3.01	3.75	0.793	0.756	0.604	0.587	0.028
PR (4)	AP31679	20	4	2.54	4.00	0.635	0.328	0.606	0.450	0.263
	AP31733	20	4	2.80	4.00	0.700	0.332	0.644	0.550	0.149
	AP31640	20	3	1.30	3.00	0.433	0.327	0.232	0.250	-0.080
	AP10368	20	6	3.17	6.00	0.528	0.086	0.686	0.200	0.714
	Mean	20	4.25	2.45	4.25	0.574	0.268	0.542	0.363	0.261
BJ (5)	AP31679	20	3	2.17	3.00	0.723	0.900	0.540	0.400	0.264
	AP31733	20	4	1.55	4.00	0.388	0.111	0.354	0.000	1.000
	AP31640	20	3	1.81	3.00	0.603	0.327	0.447	0.550	-0.237
	AP10368	20	5	5.00	5.00	1.000	0.900	0.814	0.050	0.940
	Mean	20	3.75	2.72	3.75	0.678	0.559	0.539	0.250	0.491
CGB (6)	AP31679	20	4	2.00	4.00	0.500	0.086	0.500	0.450	0.102
	AP31733	20	4	2.62	4.00	0.655	0.328	0.619	0.350	0.441
	AP31640	20	2	2.00	2.00	1.000	0.900	0.508	0.900	-0.810
	AP10368	20	6	2.12	6.00	0.353	0.043	0.528	0.500	0.055
	Mean	20	4.00	2.19	4.00	0.627	0.339	0.539	0.550	-0.053
CGS (7)	AP31679	20	4	2.75	4.00	0.688	0.280	0.637	0.750	-0.183
	AP31733	20	3	2.24	3.00	0.747	0.900	0.555	0.400	0.285
	AP31640	20	2	2.00	2.00	1.000	0.900	0.508	0.800	-0.600
	AP10368	20	7	2.86	7.00	0.409	0.026	0.651	0.300	0.546
	Mean	20	4.00	2.47	4.00	0.711	0.526	0.588	0.563	0.012
TR (8)	AP31679	20	5	3.83	5.00	0.766	0.355	0.739	0.650	0.124
	AP31733	20	3	1.45	3.00	0.483	0.334	0.311	0.250	0.202
	AP31640	20	3	2.04	3.00	0.680	0.900	0.511	0.800	-0.587
	AP10368	20	6	4.27	6.00	0.712	0.350	0.767	0.150	0.808
	Mean	20	4.25	2.90	4.25	0.660	0.484	0.582	0.463	0.137
ZGB (9)	AP31679	20	3	2.62	3.00	0.873	0.900	0.619	0.450	0.278

	AP31733	20	5	3.55	5.00	0.710	0.302	0.719	0.300	0.589
	AP31640	20	3	1.37	3.00	0.457	0.350	0.273	0.300	-0.101
	AP10368	20	8	4.01	8.00	0.501	0.047	0.751	0.300	0.607
	Mean	20	4.75	2.89	4.75	0.635	0.399	0.591	0.337	0.343
SU (10)	AP31679	20	4	2.38	4.00	0.595	0.313	0.581	0.450	0.230
	AP31733	20	3	1.62	3.00	0.540	0.297	0.383	0.050	0.872
	AP31640	20	4	2.07	4.00	0.518	0.323	0.517	0.650	-0.267
	AP10368	20	6	5.14	6.00	0.857	0.900	0.806	0.750	0.072
	Mean	20	4.25	2.80	4.25	0.627	0.458	0.572	0.475	0.226
VO (11)	AP31679	20	3	2.31	3.00	0.770	0.900	0.568	0.550	0.032
	AP31733	20	2	2.00	2.00	1.000	0.900	0.508	0.400	0.216
	AP31640	20	3	1.80	3.00	0.600	0.329	0.445	0.550	-0.244
	AP10368	20	9	5.74	9.00	0.638	0.044	0.827	0.400	0.523
	Mean	20	4.25	2.96	4.25	0.752	0.543	0.587	0.475	0.131
VP (12)	AP31679	20	3	2.60	3.00	0.867	0.900	0.617	0.600	0.028
	AP31733	20	3	2.06	3.00	0.687	0.900	0.514	0.550	-0.072
	AP31640	20	2	1.35	2.00	0.675	0.900	0.261	0.200	0.240
	AP10368	20	7	4.97	7.00	0.710	0.109	0.800	0.500	0.381
	Mean	20	3.75	2.75	3.75	0.734	0.702	0.548	0.463	0.160
KG (13)	AP31679	20	4	2.65	4.00	0.662	0.344	0.623	0.650	-0.044
	AP31733	20	3	2.12	3.00	0.707	0.900	0.529	0.100	0.815
	AP31640	20	3	1.99	3.00	0.663	0.359	0.499	0.750	-0.524
	AP10368	20	5	2.75	5.00	0.550	0.128	0.637	0.400	0.378
	Mean	20	3.75	2.38	3.75	0.645	0.432	0.572	0.475	0.156
LE (14)	AP31679	20	6	2.74	6.00	0.457	0.023	0.636	0.650	-0.023
	AP31733	20	5	4.79	5.00	0.958	0.900	0.792	0.300	0.627
	AP31640	20	4	2.31	4.00	0.578	0.331	0.568	0.750	-0.332
	AP10368	20	6	4.15	6.00	0.692	0.347	0.760	0.400	0.480
	Mean	20	5.25	3.50	5.25	0.671	0.400	0.689	0.525	0.188
MAG (15)	AP31679	20	4	2.04	4.00	0.510	0.337	0.511	0.400	0.223
	AP31733	20	7	6.85	7.00	0.979	0.900	0.855	0.700	0.185
	AP31640	20	3	2.28	3.00	0.760	0.900	0.563	0.800	-0.437
	AP10368	20	7	2.61	7.00	0.373	0.019	0.618	0.450	0.277
	Mean	20	5.25	3.45	5.25	0.656	0.539	0.637	0.588	0.061

N- number of individuals; A_N – number of alleles; A_E – number of effective alleles; AR – allelic richness; R – the ratio of effective and detected number of alleles; p(R) – statistical significance at $p<0.05$; H_E – expected heterozygosity; H_O – observed heterozygosity; F – inbreeding coefficient; P – private allele detected

Supplementary Table S2. The pF_{ST} genetic differentiation matrix between 15 populations of *Alyssum bosniacum*. Population codes correspond to those in Table 4. *Tetraploid population.

	MAG	KG	LE	VO	VP	SU	BJ	CGB	TR	ZGB	VRA	CGS	PR	VI
KG	0.1219													
LE	0.0328	0.0781												
VO	0.0808	0.0442	0.0523											
VP	0.1088	0.0750	0.0827	0.0062										
SU	0.1892	0.1249	0.1248	0.0719	0.0559									
BJ	0.1632	0.1306	0.1065	0.0460	0.0326	0.0091								
CGB	0.2055	0.1690	0.0951	0.1331	0.1965	0.2137	0.1632							
TR	0.1903	0.1641	0.1374	0.0864	0.0735	0.0497	0.0458	0.2722						
ZGB	0.1273	0.1022	0.0902	0.0553	0.0543	0.0679	0.0617	0.1938	0.1463					
VRA	0.3009	0.2445	0.1812	0.1861	0.2025	0.1735	0.1578	0.2284	0.0769	0.2610				
CGS	0.2674	0.1566	0.1969	0.1720	0.1846	0.1260	0.2501	0.3221	0.2250	0.1600	0.2806			
PR	0.1581	0.1150	0.1236	0.0839	0.0565	0.1239	0.1770	0.3025	0.1208	0.1194	0.2286	0.0987		
VI	0.2292	0.1691	0.1854	0.1022	0.0557	0.0288	0.0512	0.3086	0.0341	0.1275	0.1386	0.1680	0.0927	
JH*	0.2681	0.1393	0.1930	0.1325	0.1265	0.0562	0.0834	0.2993	0.0369	0.1799	0.1356	0.1908	0.1536	0.0560

Supplementary Table S3. Differences observed in concatenated sequences of *rpl32-trnL* and *rpoB-trnC* on the studied samples of *Alyssum bosniacum*. H1-17 correspond to the detected haplotypes.

Consensus	G	T	G	T	A	A	G	C	T	A	C	A	A	A	A	A	G	A	G	T	A	G	C	C	T	T	A	A
H1	.	.	.	.	T	.	.	.	G	.	.	.	.	.	.	C	T	.	.	.	.	.	.	.	.	.	.	.
H2	.	.	.	C	.	.	T	.	.	.	T	.	.	.	.	.	.	C	T	A	.	.	.	T	.	.	C	.
H3	.	G	A	C	.	.	T	.	.	.	.	.	C	C	T	.	.	C	.	.	.	.	.	.	.	.	C	G
H4	.	G	A	C	.	.	T	.	.	.	.	C	C		T	.	.	C	.	.	.	.	.	.	.	.	C	G
H5	.	G	A	C	.	.	T	.	.	.	.	.	C		T	.	.	C	.	.	.	.	.	.	.	.	C	G
H6	.	.	.	.	.	.	.	.	.	.	.	.	.	.	.	.	.	.	.	.	.	.	.	.	.	.	.	.
H7	.	.	.	.	T		.	.	.	.	.	.	.	.	.	.	.	.	.	.	.	.	.	.	.	.	.	.
H8	.	.	.	.	.	.	.	A	.	.	.	.	.	.	.	.	.	.	.	.	.	T	.	.	.	.	.	.
H9	.	.	.	.	.	.	.	.	.	.	.	.	.	.	.	.	.	.	.	.	.	.	T	.	.	.	.	.
H10	.	.	.	C	.	.	T	.	.	.	.	.	.	.	.	.	.	C	.	.	.	.	.	.	G	.	C	G
H11	.	.	.	G	.	.	.	.	.	.	.	.	.	.	.	.	.	.	.	.	.	.	.	.	.	.	.	.
H12	.	.	.	G	T	.	.	.	.	.	.	.	.	.	.	.	.	.	.	.	.	.	.	.	.	.	.	.
H13	.	.	.	.	T	.	.	.	G	.	.	.	.	.	.	.	.	.	.	.	.	.	.	.	.	.	.	.
H14	.	.	.	.	.	T	.	.	.	.	.	.	.	.	.	.	.	.	.	.	.	.	.	.	.	.	.	.
H15	.	.	.	.	.	.	.	.	.	.	.	.	.	.	.	.	.	.	.	.	.	.	.	.	.	C	.	.
H16	.	G	A	C	.	.	T	.	.	.	.	.	.	.	.	.	.	C	.	.	.	.	.	.	.	.	C	G
H17	A	.	.	C	.	.	T	.	.	T	.	.	.	.	.	.	.	C	.	.	C	.	.	.	.	.	C	G

Supplementary Table S4. Primer sequences from Sobczyk et al. (2017) used in this study. *Loci amplified in *A. bosniacum*; N_A- number of alleles amplified in *A. serpyllifolium*; N_A* - number of alleles amplified in *A. bosniacum*

STR loci	Primer sequence 5' → 3'	Colour	Range (bp)	N_A	Range (bp)	N_A*
AP31733*	F: CCGATTCCCAAAGATCCCGTG<br>R: GGTATTGCCGCAAAGTTTTTCA	6-FAM	187–202	5	163–187	8
AP32282	F: TCCTCCTCACTTTCGCTGAA<br>R: GACGGTTGATGGCGGTTTTG	6-FAM	294–315	5	-	-
AP31679*	F: TCCTCACCAAAGCTCAGCG<br>R: CTTAGCCTCCTCCTCCCTCT	HEX	363–399	12	291–339	8
AP801	F: TGGAGGTGGGATATGAGCAAA<br>R: CGAGCAGAGGAGACCAAAGA	HEX	208–229	5	-	-
AP31640*	F: CGAAAACTCTGCGATGTGGC<br>R: CGTCTCTTGGGTTTTGCTGC	HEX	253–292	10	231–346	6
AP34461	F: AAGGGAAAGTCAGAAGCAGAGC<br>R: AGTTCTTCAAAGTTTCATAGACAACA	6-FAM	147–168	7	-	-
AP10368*	F: TCTAACTGACGGAGGGGTTG<br>R: TGCCATTCTTGAAGCACTGC	6-FAM	343–355	4	349–388	13
AP5386	F: GGGATTCTTGGTCGGCTCAA<br>R: CTGATGGAGATGCTTGTGGGT	HEX	373–385	5	-	-

Supplementary Figure S1. Principal Coordinates Analysis (PCoA) based on microsatellite genotype variants.

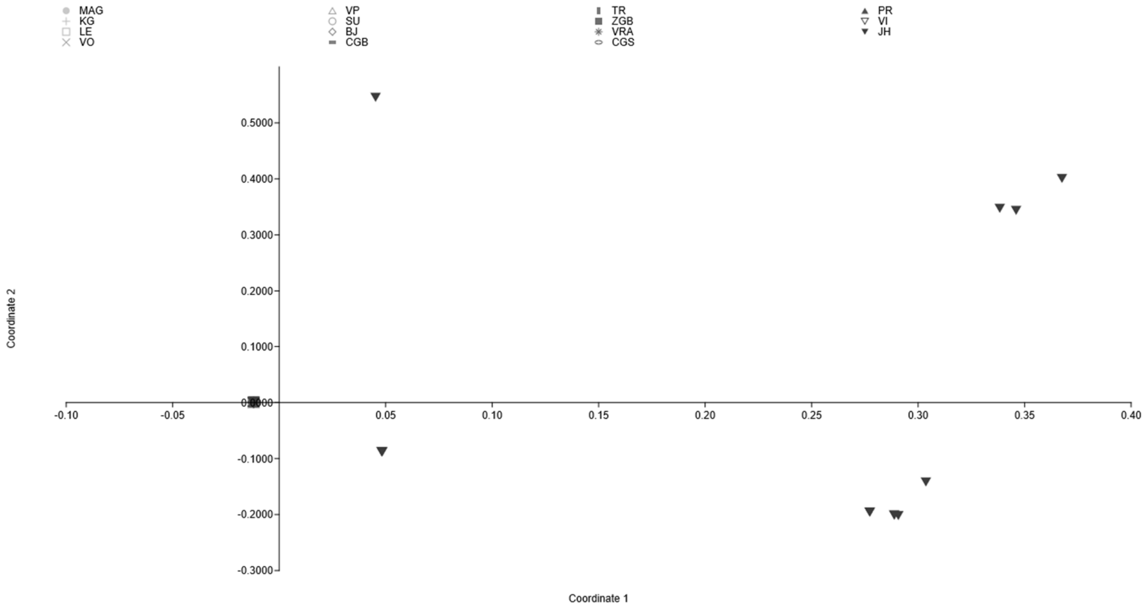

Supplementary Figure S2. Neighbor-joining (NJ) tree based on p-distance estimates for concatenated chloroplast haplotypes of *Alyssum bosniacum*, constructed using 1,000 bootstrap replicates. Bootstrap values ≥50% are shown at the corresponding nodes. Population codes correspond to those in Table 4.

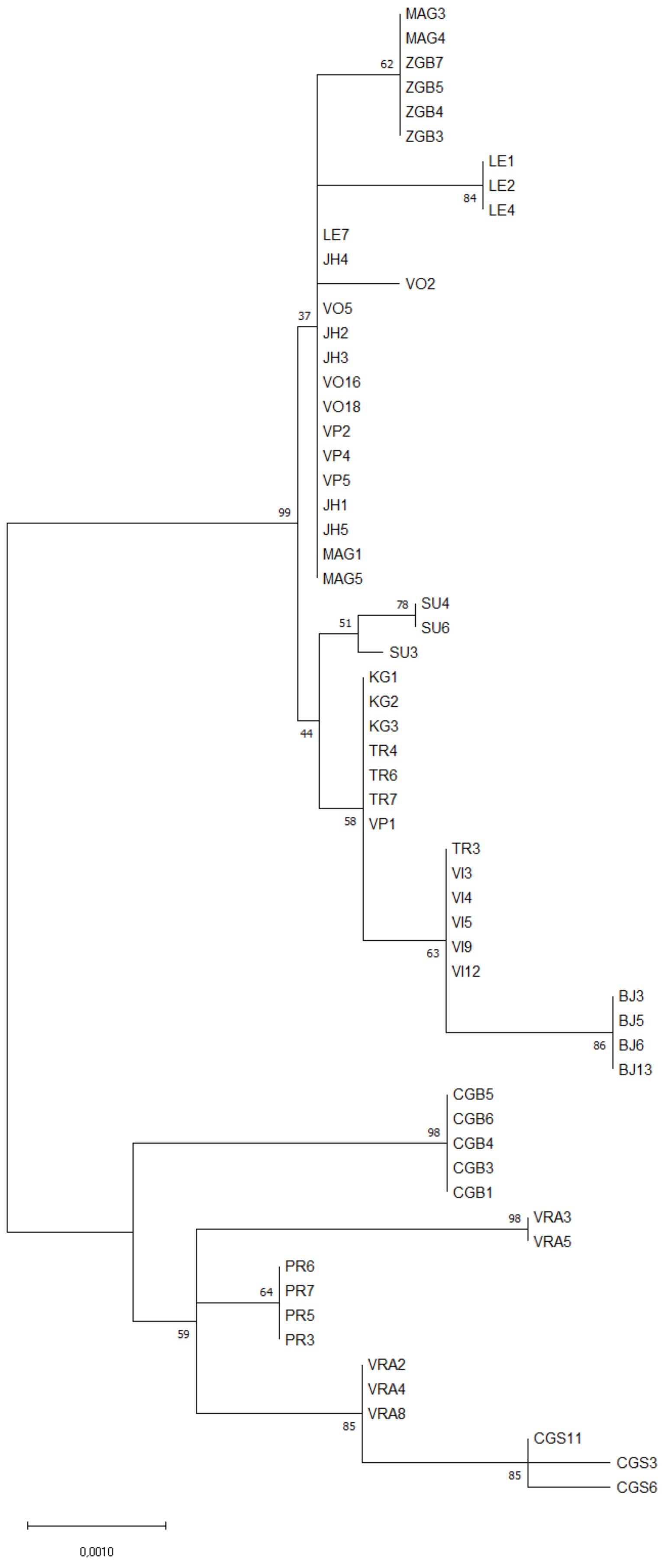
MAG3
MAG4
ZGB7
ZGB5
ZGB4
ZGB3
62
LE1
LE2
LE4
84
LE7
JH4
VO2
VO5
JH2
JH3
VO16
VO18
VP2
VP4
VP5
JH1
JH5
MAG1
MAG5
37
99
SU4
SU6
78
SU3
51
KG1
KG2
KG3
TR4
TR6
TR7
VP1
58
44
TR3
VI3
VI4
VI5
VI9
VI12
63
BJ3
BJ5
BJ6
BJ13
86
CGB5
CGB6
CGB4
CGB3
CGB1
98
VRA3
VRA5
98
PR6
PR7
PR5
PR3
64
59
VRA2
VRA4
VRA8
85
CGS11
CGS3
CGS6
85
0,0010
